# Levosimendan combined with epinephrine improves rescue outcomes in a rat model of lipid-based resuscitation from bupivacaine-induced cardiac arrest

**DOI:** 10.1186/s12871-017-0414-3

**Published:** 2017-09-15

**Authors:** Fubei Nan, Xixi Cai, Yingchao Ye, Xuzhong Xu, Zhengqian Li, Min Li, Limei Chen

**Affiliations:** 1 Department of Anaesthesiology, the First Affiliated Hospital, Wenzhou Medical University, Zhejiang, China; 2 Department of Anaesthesiology, Peking University Third Hospital ( PUTH ), No.49.North Garden Street, Haidian District, Beijing, China

**Keywords:** Bupivacaine, Cardiac arrest, Lipid emulsion, Epinephrine, Levosimendan

## Abstract

**Background:**

The effectiveness of a combination of a lipid emulsion with epinephrine in reversing local anesthetic-induced cardiac arrest has been confirmed. The combination of a lipid emulsion with levosimendan, was shown to be superior to administration of a lipid emulsion alone with regard to successful resuscitation. In this study, we compared the reversal effects of levosimendan, epinephrine, and a combination of the two agents in lipid-based resuscitation in a rat model of bupivacaine-induced cardiac arrest.

**Methods:**

Fifty-four adult male Sprague-Dawley rats were subjected to bupivacaine (15 mg·kg^−1^) –induced asystole and were then randomly divided into 3 groups. A lipid emulsion was used as the basic treatment, and administration of drug combinations varied in each group as follows: (1) levosimendan combined with epinephrine (LiEL); (2) epinephrine (LiE); and (3) levosimendan (LiL). The resuscitation outcomes were recorded and included the rate of return of spontaneous circulation (ROSC) and survival at 40 min, time to first heartbeat, time to ROSC, and cumulative dose of epinephrine. We calculated the wet-to-dry ratio of the lung, blood gas values at 40 min and bupivacaine concentration of cardiac tissue and plasma.

**Results:**

The rates of ROSC in LiEL and LiE groups were higher than LiL group (*P* < 0.001; LiEL vs LiL, *P* = 0.001; LiE vs LiL, *P* = 0.007). The survival rate in LiEL group was higher than LiE group (*P* = 0.003; LiEL vs LiE, *P* = 0.008; LiEL vs LiL, *P* = 0.001). The time to first heart beat in LiEL group was shorter than LiE, LiL groups. (*P* < 0.001; LiE vs LiEL, *P* = 0.001; LiL vs LiEL, *P* < 0.001). The time to ROSC in LiEL group was shorter than LiE, LiL groups (*P* < 0.001; LiEL vs LiE, *P* < 0.001; LiEL vs LiL, *P* < 0.001). The result was similar for the bupivacaine concentration of cardiac tissue and plasma (cardiac tissue: *P* = 0.002; plasma: *P* = 0.011). Furthermore, there were significant differences in the blood-gas values at 40 min, wet-to-dry lung weight ratio, and ratio of damaged alveoli among groups. The LiEL group had the best result for all parameters (*P* < 0.01, *P* = 0.008, *P* < 0.001, respectively). Additionally, significantly less epinephrine was used in the LiEL group (*P* < 0.001).

**Conclusions:**

Levosimendan combined with epinephrine may be superior to either drug alone for lipid-based resuscitation in a rat model of bupivacaine-induced cardiac arrest. The drug combination was associated with a higher survival rate as well as decreased epinephrine consumption and lung damage.

## Background

The guidelines for advanced cardiac life support that were developed by the American Heart Association in 2015 increased the emphasis on the importance of chest compressions and respiratory support [1]. Nevertheless, in the clinical cardiac arrest scenario, epinephrine is also widely used. A previous study showed that epinephrine could accelerate the recovery of cardiovascular collapse induced by a local anesthetic [2]. In our own work, we also found that the combination of a lipid emulsion with epinephrine was superior to lipid emulsion or epinephrine alone with regard to the recovery of cardiac function in an isolated rat heart model of bupivacaine-induced cardiac arrest [3]. Recently, our team confirmed that levosimendan supplemented with epinephrine could significantly enhance the survival rate in rats that experienced asphyxia-induced cardiac arrest [4]. By contrast, some other studies reported that epinephrine induced deterioration of pulmonary oxygen exchange, led to pulmonary edema and failed to improve the survival rate in lipid-based resuscitation of bupivacaine-induced cardiac arrest [5, 6]. Specifically,Hill et al [7] showed that epinephrine >10 μg·kg^−1^ impaired the efficacy of lipid resuscitation from bupivacaine overdose. The American Society of Regional Anesthesia and Pain Medicine subsequently promulgated the guidelines [8] in which a small dose of epinephrine (< 5 μg·kg^−1^) was recommended.

Levosimendan is a novel calcium sensitizer. A study reported that levosimendan could reverse the ropivacaine-induced negative inotropic effect in the isolated heart [9]. Gokahmetoglu et al [10] found that levosimendan combined with a lipid emulsion was associated with a higher survival rate compared with a lipid emulsion alone for the reversal of bupivacaine-induced cardio-toxicity.

In the present study, we hypothesized that adding epinephrine and levosimendan to a lipid emulsion enhanced resuscitation from bupivacaine-induced cardiac arrest compared with adding epinephrine or levosimendan alone. Accordingly, this double-blind prospective randomized animal study was aimed at assessing the rate of ROSC following different resuscitation protocols.

## Methods

### Experimental animals and groups

The experimental protocol was approved by the Animal Care and Use Committee of Wenzhou Medical University (Wenzhou, Zhejiang, China). Fifty-four male Sprague-Dawley rats (2-months-old; weight 300-350 g), were purchased from Shanghai Slac Laboratory Animal Co, Ltd. (Shanghai, China) and bred under standardized housing conditions. They were allowed to adapt to their new environment for at least 7 days before beginning the experiments. The rats were randomly divided into 3 groups (18 rats per group); each group received one of the following treatments after cardiac arrest was induced by a bupivacaine overdose: (1) a lipid emulsion combined with epinephrine and levosimendan (LiEL); (2) lipid emulsion and epinephrine (LiE); or (3) lipid emulsion and levosimendan (LiL).

### Experimental model

The animals were fasted for 12 h before the experiment, but had access to water ad libitum. They were anesthetized with an intraperitoneal injection of chloral hydrate (5%, 350 mg/kg) to allow intubation via tracheotomy. Rats were then ventilated with 0.5% sevoflurane in oxygen (HX-300 ventilator; TME Technology Co, Ltd., Chendu, China). The mechanical ventilation parameters were set as previously described [11]: tidal volume 8 mL/kg, respiratory rate 70–80 breaths/min, and inspiratory: expiratory ratio = 2:3. The left external jugular vein, left femoral vein, and left femoral artery were cannulated for infusion of bupivacaine (Sigma-Aldrich, St. Louis., MO, USA), lipid emulsion (Huarui Pharmaceuticals Co., Ltd., Wuxi, China), and levosimendan (Qilu Pharmaceuticals Co., Ltd., Jinan, China). The femoral artery was then connected to the MadLab data collection and processing system (Medlab-U/4C051; Nanjing Medease Science and Technology Co., Ltd., Jiangsu, China) to continuously record the arterial pressure. After the invasive operations, rats were allowed to stabilize for 15 mins, and then, the baseline blood gas values, systolic pressure, heart rate, and rate-pressure product (RPP) were recorded.

### Bupivacaine arrest and resuscitation protocol

At the end of the stabilization period, sevoflurane was discontinued, and 15 mg^.^kg^−1^ bupivacaine was injected as an intravenous bolus over 15 s via the venous cannula in the left femoral vein. All rats developed asystole at the end of the bupivacaine infusion, and this point was defined as time zero.

Manual external chest compressions (on the lower-middle sternum at 300 compressions per min and a depth of 1 cm) were instituted immediately upon the onset of cardiac arrest (time zero) until ROSC (the criterion of ROSC was regular autonomic rhythm of the heart, and the RPP was more than 20% of the baseline value for 1 min [11, 12]) or 40 min without successful resuscitation. At the same time, all animals received a lipid emulsion as a 5 mL·kg^−1^ bolus for 15 s, followed by a continuous infusion of a lipid emulsion at 1 mL·kg^−1^·min^−1^ for 5 min via the external jugular vein using a micro-infusion pump (Graseby 3500 Syringe Pump; Smith Medical International Co., Ltd., UK). This lipid therapy regimen was based on previous studies conducted by our laboratory and others with some modifications [13, 14]. After the bolus of the lipid emulsion, the three groups immediately received the resuscitation drugs. To standardize the total volume, all groups received the same volume. Group LiEL received epinephrine 2 μg·kg^−1^ (1 mL·kg^−1^) and levosimendan 150 μg·kg^−1^ (6 mL·kg^−1^); Group LiE received epinephrine 2 μg·kg^−1^ (1 mL·kg^−1^) and saline 6 mL·kg^−1^; Group LiL received saline 1 mL·kg^−1^ and levosimendan 150 μg·kg^−1^ (6 mL·kg^−1^). Rats in Group LiEL and Group LiE received repeated doses of 1 μg·kg^−1^ epinephrine at 2-min intervals until ROSC or the end of the resuscitation period of 40 min. External chest compressions were stopped when ROSC was achieved, which we defined as successful resuscitation. However, in animals that developed a second cardiac arrest after the initial ROSC, only chest compressions were performed without epinephrine until the termination. The temperature of the rats was maintained at 38 °C to 39 °C by an adjustable electric blanket. All drugs were prepared and preheated to 37 °C by personnel who were not involved in the subsequent resuscitation. Researchers were blinded to the group assignments. Resuscitation was performed according to the following teamwork algorithm [11]: provider 1 was responsible for the infusion of the lipid emulsion and resuscitation drugs; provider 2 implemented external chest compressions; and provider 3 maintained the airway and assessed the native RPP during resuscitation. All of the providers were blinded to the group allocations. At the end of the observation period of 40 min, a 4 mL blood sample was immediately taken from the left femoral artery of each animal for the analysis of the blood gas values and plasma bupivacaine concentration. The rats were euthanized by air injection, and tissue samples were taken from the lung and heart of each animal for further study. All samples were stored at −80 °C until analysis was performed.

### Data acquisition

ROSC and the rats that survived for 40 min were recorded. We then calculated the rates of ROSC, survival and mortality at 40 min. The SBP and HR of the survivors in the 3 groups were continuously recorded, and the RPP was calculated during the 40-min observation period. We documented the time to first heartbeat and ROSC, as well as the cumulative dose of epinephrine used.

### Lung pathology and wet-to-dry ratio

Lung samples were obtained at the termination of the observation period. After sacrifice, the middle lobe of the right lungs were excised, placed into a 10% formalin fixative, embedded with paraffin and cut into slices. Sections were stained with hematoxylin-eosin for light microscopy. Fifty views (400× power) of each sample were randomly selected. The number of total alveoli and damaged alveoli (manifestation of more than 2 inflammatory cells or 2 red blood cells in one pulmonary alveolus was defined as damaged [15]) in each view were counted, and the ratio of the damaged alveoli in each view was calculated. Additionally, left lung samples were obtained, blotted dry and weighed immediately (wet weight). The samples were then subjected to desiccation in a dryer at 70 °C for 48 h to obtain the dry weight. The wet-to-dry ratio of the lung was calculated.

### Bupivacaine measurement

For animals in each group, the bupivacaine concentrations in plasma and cardiac tissue at 40 min were analyzed as previously described [16, 17]. The liquid chromatography-tandem mass spectrometric (Bruker Esquire; Bruker Company, Karlsruhe, Germany) method was used to determine the bupivacaine concentration. The parameters of liquid chromatography-tandem mass spectrometry were the same as the parameters in Chen’s [16] report. Frozen heart tissue (0.2 g) was homogenized with 2 mL of ultrapure water. Then, 200 μL of the homogenate or plasma was added to 62.5 ng of ropivacaine as an internal standard; 0.05 M NaOH was added for alkalization, and 1 mL N-hexane and 4 mL acetoacetate was used for the extraction of bupivacaine. The compound was mixed with a vortex generator for 1 min and centrifuged at 4000 rpm for 15 min. The upper organic phase was neutralized and acidified with 0.3 mL of 1.5% HCL, mixed with a vortex generator for 1 min, and centrifuged at 4000 rpm for 15 min. A total of 200 μL of the aqueous phase subnatant was removed for the detection of bupivacaine.

### Statistical analysis

Power analysis was based on our preliminary experiment using Power Sample Size (PASS 11) software. We compared the survival rate among the three groups. In our preliminary study, 24 rats in 3 groups were included. At the endpoint of 40 min, 7 (87.5%), 4 (50%) and 2 (25%) rats survived in the LiEL, LiE, and LiL groups, respectively. The Power was set at 0.8. Our primary indicator was the survival rate, and the significance criterion was divided by 3, which was set at 0.017. Thus, 16 rats were needed per group to achieve statistical significance between the LiEL and LiE groups as well as between the LiEL and LiL groups. To account for the potential attrition, we enrolled 18 rats per group.

All data were analyzed with SPSS 17.0 statistical software. The normal distribution was tested using the Shapiro-Wilk test. Measurement data were expressed using the means ± SD or medians and the interquartile range. The baseline values, time of the first heartbeat, blood gas values, wet-to-dry weight ratio of the lung, and concentrations of bupivacaine in plasma were compared by one-way analysis of variance. The time to ROSC, alveolar damage ratio, and concentrations of bupivacaine in cardiac tissue were compared using the Kruskal-Wallis H test. Homogeneity of variance among the three groups was determined by the least significant difference test (LSD), while heterogeneous data were determined by Dunnett’s T3 test. Continuous hemodynamic variables in animals were compared by repeated measures analysis of variance, an Bonferroni correction was used to determine the post-test scores. A *P-*value <0.05 was considered statistically significant.

## Results

### Characteristics of study subjects

The baseline values of the weight, hemodynamic metrics, and blood gas values did not differ significantly among the three groups (Table 1).

**Table 1:** Baseline Values of Weight, Hemodynamic Metrics, and Blood Gas Values

Characteristics	Group LiEL (*n* = 18)	Group LiE (*n* = 18)	Group LiL (*n* = 18)
Weight (g)	320 ± 10	316 ± 12	323 ± 16
SBP (mmHg)	130 ± 21	130 ± 16	125 ± 13
HR (Beat/Min)	399 ± 48	409 ± 37	397 ± 59
RPP (mmHg·Beat/Min)	52,105 ± 14,472	53,452 ± 9572	50,887 ± 10,975
pH	7.41 ± 0.06	7.39 ± 0.03	7.39 ± 0.05
Base excess (mmol/L)	−1.8 ± 2.3	−2.0 ± 2.2	−1.4 ± 2.0
PaO_2_ (mmHg)	298 ± 82	310 ± 73	327 ± 73
PaCO_2_ (mmHg)	36.5 ± 2.9	35.9 ± 3.6	37.0 ± 3.5
Lactate (mmol/L)	1.6 ± 0.5	1.8 ± 0.6	1.9 ± 0.5

Values are given as mean ± SD, there was no difference beteen three groups. SBP indicates systolic blood pressure. HR indicates heart rate. RPP indicates rate-pressure product (systolic blood pressure × heart rate). BE indicates base excess

### Resuscitation outcomes

A total of 18, 17 and 9 animals achieved ROSC in the LiEL, LiE and LiL groups, respectively. The rates of ROSC in LiEL and LiE groups were higher than LiL group (Table 2). A total of 6 out of the 17 ROSC rats in the LiE group experienced a second cardiac arrest. In the end, 18, 11 and 9 rats survived for 40 min, with a survival rate of 100%, 61.1%, and 50%, respectively. The survival rate in LiEL group was higher than in either LiE or LiL groups. The time to first heart beat in LiEL group was shorter than LiE, LiL groups. The time to ROSC in LiEL group was shorter than LiE, LiL groups.

**Table 2:** Resuscitation Outcomes for LiEL, LiE and LiL groups

Characteristics	Group LiEL (*n* = 18)	Group LiE (*n* = 18)	Group LiL (*n* = 18)
Rate of ROSC,%	100%^#^	94.4%^#^	50%
Survival rate,%	100%	61.1%^*^	50%^*^
Time to first heart beat (s)	44 ± 12	137 ± 85^*^	170 ± 90^*^
Time to ROSC (s)	142(120,151)	317(238,495)^*^	1590(313,2400)^*^
Epinephrine cumulative dose (μg/kg)	3 (2, 3)	6 (4, 8)^*^	_____________

Data are given as mean ± SD or medians and interquartile. The rate of ROSC in each group displayed differences (*P* < 0.001; LiEL vs LiL, *P* = 0.001; LiE vs LiL, *P* = 0.007). The LiEL, LiE, LiL groups displayed differences in survival (*P* = 0.003; LiEL vs LiE, *P* = 0.008; LiEL vs LiL, *P* = 0.001). The LiEL, LiE, and LiL groups displayed differences in time to first heart beat and ROSC (first heart beat: *P* < 0.001; LiE vs LiEL, *P* = 0.001; LiL vs LiEL, *P* < 0.001. ROSC: *P* < 0.001; LiEL vs LiE, *P* < 0.001; LiEL vs LiL, *P* < 0.001). The LiEL and LiE groups displayed differences in epinephrine cumulative dose (*P* < 0.001). ^*#*^ *P* < 0.01 vs LiL, ^*^ *P* < 0.01 vs LiEL. ROSC indicates return of spontaneous circulation

### Hemodynamic measures

**Fig. 1:**
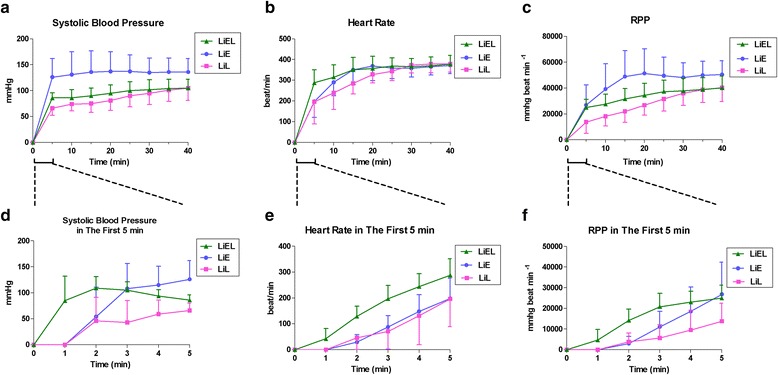
Hemodynamic parameters for rats that survived to 40 min. Systolic blood pressure (**a**), heart rats (**b**), and RPP (**c**) versus time for these survivors during the 40-min observation period were shown in the upper panel. On considering the importance of the early 5 min for the successful resuscitation, systolic blood pressure (**d**), heart rats (**e**), and RPP (**f**) were also specially shown in the lower panel. Significant differences were demonstrated in systolic blood pressure and RPP, but not in heart rate during the 40-min observation period between LiEL, LiE, and LiL groups (*P* < 0.001; *P* = 0.001; *P* = 0.128). Importantly, there were significant differences in above three parameters between LiEL, LiE, and LiL groups in the first 5 min (*P* < 0.001 for each). Data are presented as means and SD. SBP indicates systolic blood pressure; HR indicates heart rate; RPP indicates rate-pressure product (systolic blood pressure × heart rate)

Hemodynamic parameters including the systolic blood pressure, heart rate and RPP (Fig. 1) of the animals that survived to the endpoint of 40 min are presented graphically. The LiEL, LiE, and LiL groups displayed differences in the hemodynamic parameters. We primarily compared the parameters across the three groups at an early point (5 min) of the observation period. These hemodynamic measures displayed significant differences throughout the 40-min observation period among the LiEL, LiE and LiL groups. As shown in the lower panel of Fig. 1, there were significant differences in the above three parameters among the three groups within the first 5 min (*P* < 0.001 for each).

### Blood gas analysis

The arterial blood gas values of all rats at 40 min are presented in Table 3. pH in LiEL group was higher than LiE and LiL groups; The PaCO_2_ in LiEL group was lower than LiL group; The BE in LiEL group was lower than LiE group and higher than LiL group; The PaO_2_ in LiEL group was higher than LiE and LiL groups; Lactate in LiEL group was lower than LiE and LiL groups.

**Table 3:** Arterial blood-gas values at 40 min

Characteristics	Group LiEL (*n* = 18)	Group LiE (*n* = 18)	Group LiL (*n* = 18)
pH	7.41 ± 0.05	7.11 ± 0.28^*^	7.08 ± 0.30^*^
PaCO_2_ (mmHg)	38 ± 8	53 ± 24	62 ± 25^*^
BE (mmol/L)	−1.6 ± 2.0	11.3 ± 9.8^*^	−10 ± 8.7^*^
PaO_2_ (mmHg)	266 ± 101	140 ± 89^*^	123 ± 81^*^
Lactate (mmol/L)	2.2 ± 0.7	5.1 ± 4.0^*^	6.2 ± 4.7^*^

Data are given as mean ± SD. The LiEL, LiE, LiL groups displayed differences in PH (*P* < 0.001; LiEL vs LiE, *P* = 0.001; LiEL vs LiL, *P* = 0.001); The LiEL, LiE, LiL groups displayed differences in PaCO_2_ (*P* < 0.001; LiEL vs LiL, *P* = 0.003); The LiEL, LiE, LiL groups displayed differences in BE(*P* = 0.001; LiEL vs LiE, *P* = 0.002; LiEL vs LiL, *P* = 0.002); The LiEL, LiE, LiL groups displayed differences in PaO_2_ (*P* < 0.001; LiEL vs LiE, *P* < 0.001; LiEL vs LiL, *P* < 001); The LiEL, LiE, LiL groups displayed differences in LAC (*P* = 0.008; LiEL vs LiE, *P* = 0.026; LiEL vs LiL, *P* = 0.008). ^*^ *P* < 0.05 vs LiEL. BE indicates base excess

### Wet-to-dry ratio

The wet-to-dry ratio (*n* = 18, in all groups) was significantly different among the three groups (4.9 ± 0.4, 5.5 ± 0.7, and 5.3 ± 0.3, for the LiEL, LiE, LiL groups, respectively, *P* = 0.008). The wet-to-dry ratio of the LiEL group was significantly lower than that of the LiE group (*P* = 0.002).

### Lung pathology examination

**Fig. 2:**
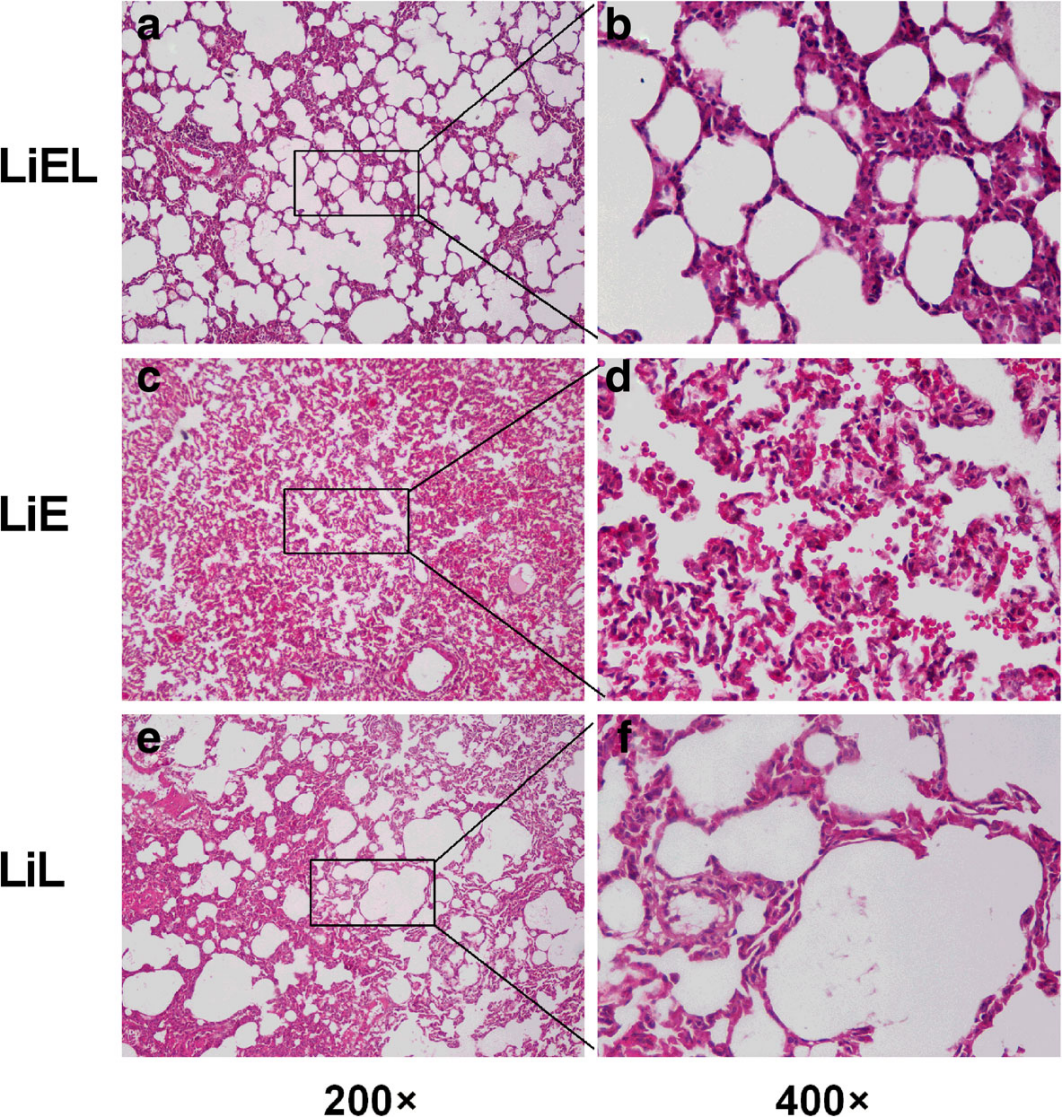
The light microscopy view of the right middle lobe of survivors in the group LiEL (**a**, **b**), LiE (**c**, **d**), and LiL (**e**, **f**). For the rats in group LiEL, the structures of alveoli were generally normal, and no leukocytes or erythrocytes were observed. In group LiE, most of the alveoli were destroyed as evidenced by the almost changed structure. Furthermore, there was erythrocytes infiltration in the damaged alveoli. For the survivors in group LiL, some normal alveoli still existed and several erythrocytes were observed. Magnification: 200× (left panel), 400× (right panel)

The microscopic views of the structure of the pulmonary alveoli in the LiEL group were normal. Edema and hemorrhage were rarely observed, and the alveolar walls were intact. Neither pulmonary interstitial edema nor leukocytes/erythrocytes was observed. In the other two groups, the structure of several alveoli was damaged. In the alveoli and pulmonary interstitium, moderate-to-severe leukocyte infiltration was observed. The alveolar damage was most obvious in the LiE group (Fig. 2).

There was a significant difference in the number of damaged alveoli among the LiEL [10% (5%, 14%)], LiE [88% (79%, 96%)], and LiL [56% (48%, 75%)] groups. The rate of damaged alveoli in the LiEL group was significantly lower than the other two groups (*P* < 0.001).

### Bupivacaine measurement

**Fig. 3:**
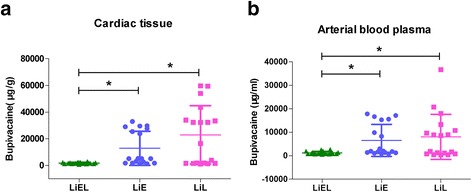
The concentration of bupivacaine in cardiac tissue (**a**) and plasma (**b**). The concentration of bupivacaine in cardiac tissue in LiEL, LiE, and LiL groups were 1722 (1307, 2132) ng/g, 5115 (1811, 28,002) ng/g, 24,389 (1683, 36,474) ng/g, respectively. Significant differences were demonstrated in three groups (P = 0.002; LiEL vs. LiE, *P* = 0.001; LiEL vs. LiL, P = 0.007). The plasma bupivacaine concentration in LiEL, LiE, and LiL groups were (1278 ± 619) ng/mL, (6560 ± 6818) ng/mL, and 8079 ± 9614) ng/mL, respectively. They displayed differences in three groups (*P* = 0.011; LiEL vs. LiL, *P* = 0.024; LiEL vs. LiE, *P* = 0.013). *P < 0.05. Data were means and SD or medians and interquartile

The concentrations of bupivacaine in cardiac tissue and plasma are presented in Fig. 3. The concentrations of bupivacaine in cardiac tissue and plasma in the LiEL group were significantly lower than in the other two groups (cardiac: *P* = 0.002; LiEL vs. LiE, *P* = 0.001; LiEL vs. LiL, *P* = 0.007. plasma: *P* = 0.011; LiEL vs. LiL, *P* = 0.024; LiEL vs. LiE, *P* = 0.013).

## Discussion

Our study demonstrated that rats receiving levosimendan and epinephrine after a lipid emulsion bolus had the highest survival rate and lowest concentrations of bupivacaine in cardiac tissue and plasma. Histologic examination of the lung was in concordance with these results, displaying decreased pulmonary hemorrhage in the levosimendan and epinephrine group (LiEL). Additionally, the LiEL group required less epinephrine than the LiE group.

Hiller et al [7] found that in an in vitro rat model, when >10 μg·kg^−1^ epinephrine was administered with a lipid emulsion, serious pulmonary edema and acidosis occurred, and the resuscitation was compromised. The survival rate of animals receiving 1 μg·kg^−1^ or 2.5 μg·kg^−1^ epinephrine was higher than those receiving 10 μg·kg^−1^. Based on the results of this study, the initial dose of 2 μg·kg^−1^ epinephrine administered at 2-min intervals was chosen for the present study. This time interval was chosen because the half-life of epinephrine is 1 min [18]. The dose of bupivacaine was 15 mg·kg^−1^, which is in accordance with our previous study [13], and rats would not survive without resuscitation. In our preliminarily experiment, we found that the dose of 150 μg·kg^−1^ of levosimendan was associated with the highest survival rate; therefore, this dose was chosen for the stusy.

In the reversal of bupivacaine-induced asystole, we previously demonstrated that [3] in an isolated rat heart, a lipid emulsion combined with epinephrine resulted in better recovery of cardiac function than a lipid emulsion or epinephrine alone. In addition, Harvey et al [19] demonstrated in a study examining the role of epinephrine in lipid-based resuscitation from bupivacaine-induced cardiac arrest that epinephrine seemed to be necessary for ROSC. All of these studies indicated that epinephrine was essential for lipid resuscitation in bupivacaine-induced cardiac arrest. However, Weinberg et al [14] demonstrated that adding epinephrine to a lipid emulsion could cause severe pulmonary edema in bupivacaine-induced cardiac arrest. Additionally, the present study suggested that if great amounts of epinephrine were used, more serious pulmonary edema and worse pulmonary oxygenation would occur. A possible explanation is that bupivacaine inhibits cardiac function and epinephrine causes intense peripheral vascular contraction, which leads to acute left heart failure as evidenced by the increased wet-to-dry lung weight ratio and decreased pulmonary partial pressure of oxygen in the LiE group vs. the LiEL group.

Levosimendan, a new calcium sensitizer, reinforces myocardial contractility without increasing the intracellular calcium concentration; therefore, it will not increase myocardial oxygen demand or lead to arrhythmias [20]. One study reported that levosimendan increased coronary perfusion flow and myocardial blood flow and strengthened the contractility of the heart in decompensated heart failure patients who received levosimendan therapy [21]. In one study [22] that used a cardiac arrest model of pigs subject to ventricular fibrillation, the use of levosimendan combined with epinephrine resulted in a higher coronary perfusion pressure than epinephrine alone. Xanthos et al [23] reported that compared with epinephrine alone, levosimendan combined with epinephrine led to a better cardiac function after resuscitation. In our study, because of the presence of levosimendan, the bupivacaine concentration of the LiEL group was significantly lower than that of the other two groups. Levosimendan has vasodilatory properties, which increases coronary artery flow [21]. Adding levosimendan to epinephrine could enhance the potency of epinephrine’s effect on ventricular contractility and coronary dilation during acidosis [24], which provides more blood and oxygen to the myocardium. Thus the heartbeat in the LiEL group was restored earlier, and the cardiac bupivacaine concentration decreased more promptly. Meanwhile, the early recovery of the heartbeat promoted the recovery of hemodynamics as manifested in the higher values of SBP, HR and RPP in the LiEL group compared with the other two groups in the early resuscitation stage. Thus, the rate of metabolism increased, and the bupivacaine concentration in plasma decreased.

De Witt et al [25] reported that levosimendan could reduce elevated pulmonary artery pressure. Morelli et al [26] indicated that in patients with acute respiratory distress syndrome, levosimendan could lower the afterload of the heart. In bupivacaine-induced cardiac toxicity, the intense contractility effect of epinephrine increased the afterload of the heart, which could exacerbate the inhibitory effect of local anesthetics. The pulmonary venous pressure could increase dramatically, which would lead to pulmonary hemorrhage and edema. The vasodilatory effect of levosimendan leads to vasodilation of peripheral resistance vessels and a decrease in both afterload and preload, thus countering the side effect of epinephrine to a certain extent and alleviating the damage to the lungs. In our study, the milder alveolar damage in the LiEL group compared with the other two groups could explain why the rats in the LiEL group had a higher survival rate.

Our investigation has several limitations. First, we assessed pulmonary edema by using the wet-to-dry ratio, and the pathology examination was performed to reflect alveolar damage. Monitoring by the combined application of other reliable parameters, including the coronary pressure, coronary arterial blood flow, and pulmonary vascular pressure, during resuscitation may be more convincing. Second, the dosing protocols of levosimendan and epinephrine that we used may not be optimal. Further research on this issue is warranted in other humanized animal models.

## Conclusion

When levosimendan combined with epinephrine was added to the basic lipid emulsion used in resuscitation of bupivacaine-induced cardiac arrest, the results demonstrate that there was a higher survival rate, better blood gas values, lower cardiac and plasma bupivacaine concentrations, and attenuated lung damage compared with administration of epinephrine or levosimendan alone.

## Abbreviations

BE: Base excess
HR: Heart rate
ROSC: Return of spontaneous circulation
RPP: Rate-pressure product
SBP: Systolic Blood Pressure
